# A secondary abdominal aorta-duodenal fistula accompanied with acquired Immune Deficiency Syndrome presented with recurrent sepsis: a case report

**DOI:** 10.1186/s12879-024-09559-8

**Published:** 2024-07-04

**Authors:** Xianjin Hu, Libo Yan

**Affiliations:** 1 Department of Cardiology, West China Hospital of Sichuan University, Chengdu, Sichuan Province China; 2 Center of Infectious Diseases, West China Hospital of Sichuan University, Chengdu, Sichuan Province China

**Keywords:** Secondary abdominal aorta-duodenal fistula, Salmonella, AIDS, In situ aorta repairment, Case report

## Abstract

**Background:**

Abdominal aorta-duodenal fistulas are rare abnormal communications between the abdominal aorta and duodenum. Secondary abdominal aorta-duodenal fistulas often result from endovascular surgery for aneurysms and can present as severe late complications.

**Case presentation:**

A 50-year-old male patient underwent endovascular reconstruction for an infrarenal abdominal aortic pseudoaneurysm. Prior to the operation, he was diagnosed with Acquired Immune Deficiency Syndrome and Syphilis. Two years later, he was readmitted with lower extremity pain and fever. Blood cultures grew *Enterococcus faecium*, *Salmonella*, and *Streptococcus anginosus*. Sepsis was successfully treated with comprehensive anti-infective therapy. He was readmitted 6 months later, with blood cultures growing *Enterococcus faecium* and *Escherichia coli*. Although computed tomography did not show contrast agent leakage, we suspected an abdominal aorta-duodenal fistula. Esophagogastroduodenoscopy confirmed this suspicion. The patient underwent in situ abdominal aortic repair and received long-term antibiotic therapy. He remained symptom-free during a year and a half of follow-up.

**Conclusions:**

This case suggests that recurrent infections with non-typhoidal Salmonella and gut bacteria may be an initial clue to secondary abdominal aorta-duodenal fistula.

## Background

**Fig. 1:**
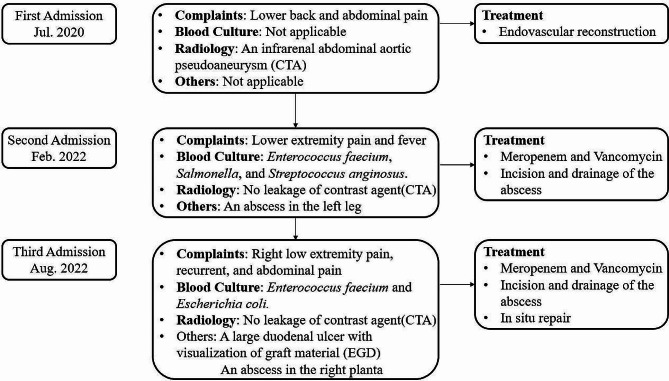
Flow chart

Secondary ADFs (SADFs) are often caused by the endovascular surgery for aneurysms, presenting as a severe late complication with an incidence ranging from 0.77–1.6% [1]. In this case, we described the diagnosis, treatment and follow-up of an SADF patient who presented with recurrent sepsis and lower extremity abscess (Fig. 1). It was noteworthy that the patient also had Acquired Immune Deficiency Syndrome (AIDS) and syphilis, which may increase the risk of infection and arterial damage. Informed consent was obtained from the patient.

## Case presentation

### First admission

**Fig. 2:**
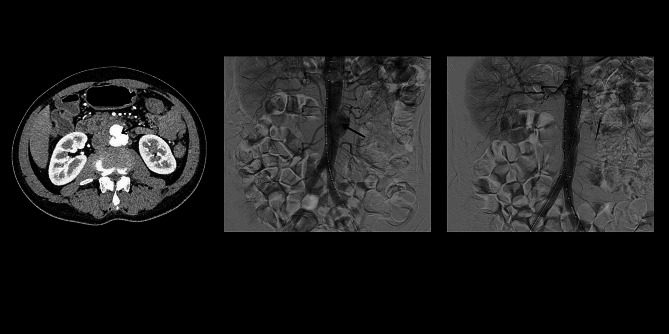
The left image depicts an infrarenal abdominal aortic pseudoaneurysm (1.7 cm × 3.4 cm) at the L2/3 level as seen on CTA. The middle image shows the pseudoaneurysm before endovascular treatment via angiography. The right image demonstrates the absence of contrast agent leakage peri-stent after the operation

**Fig. 3:**
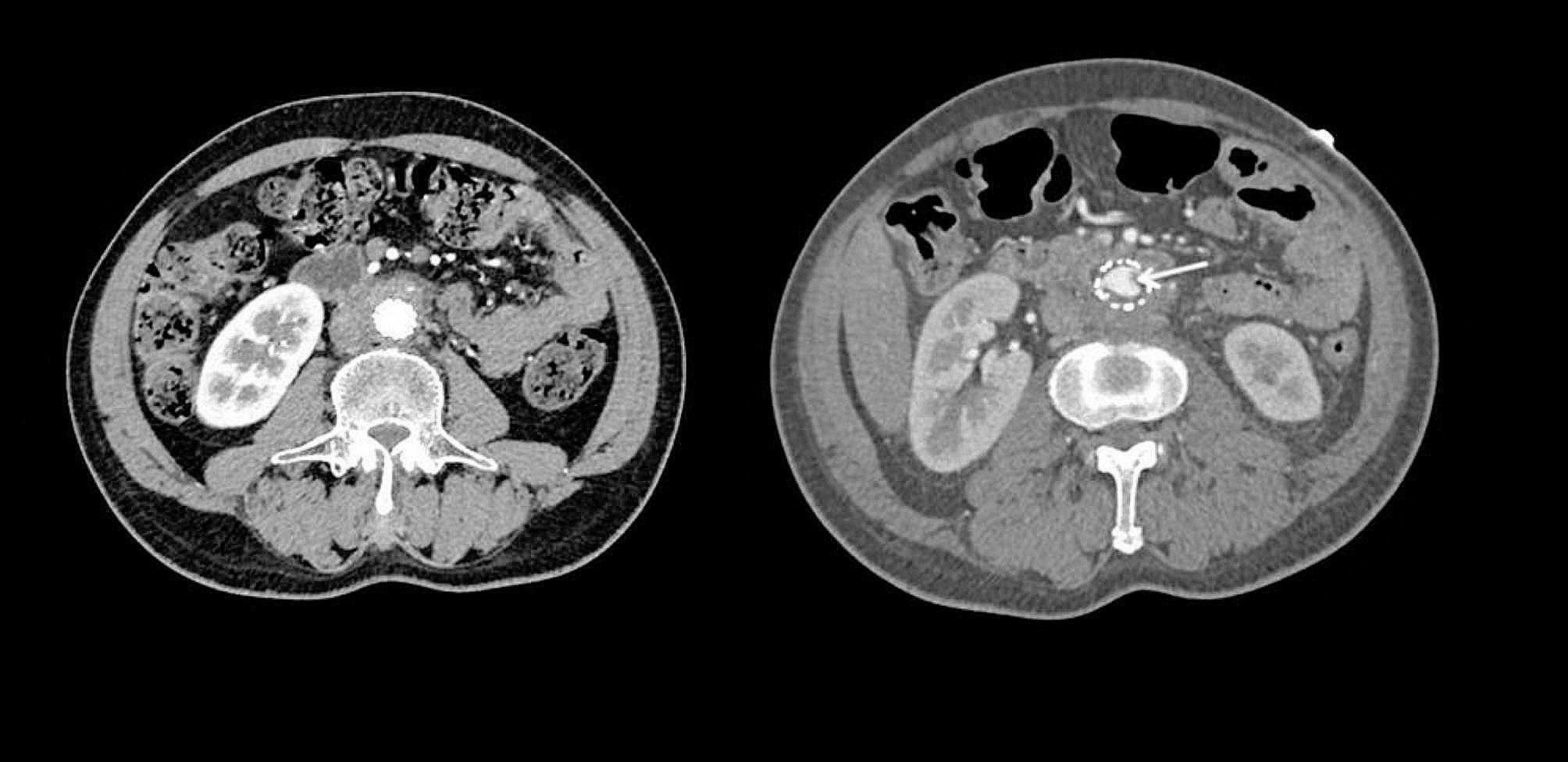
The left image shows the first rechecked CTA on August 27th, 2020. The right image shows intra-stent mural thrombosis and peri-stent soft tissue shadow

A 50-year-old man presented to the emergency department of our hospital on July 24th, 2020, with a one-week history of lower back and abdominal pain that worsened over the past day. Physical examination was unremarkable except for periumbilical tenderness. Computed Tomography Angiography (CTA) revealed an infrarenal abdominal aortic pseudoaneurysm (1.7 cm × 3.4 cm) at the L2/3 level (Fig. 2). He underwent endovascular reconstruction using two stents (Medtronic ETEW2020C82EE, Diameter: 20 mm, Coating length: 82 mm; Medtronic ETCF2323C49EE, Diameter: 23 mm, Coating length: 49 mm, Bare length: 15 mm). He was diagnosed with AIDS and syphilis before the operation. Postoperative angiography and CTA (Fig. 2) showed no leakage of contrast agent around the stent. He was treated with a combination therapy (tenofovir, lamivudine, efavirenz) for HIV. Between September 2020 to August 2020, he underwent nine treatments for syphilis. He underwent two follow-up CTA scans (August 27^ed^, 2020 and October 31st, 2021) after endovascular reconstruction, which did not show any leakage (Fig. 3).

### Second admission

The patient was readmitted on February 21st, 2022, with lower extremity pain and fever for the past ten days. He had a highest recorded temperature of 40℃ accompanied by shivering and chills. Blood cultures grew *Enterococcus faecium*, *Salmonella*, and *Streptococcus anginosus*. CTA revealed intra-stent mural thrombosis and peri-stent soft tissue shadow, with no sign of leakage (Fig. 3). Ultrasonography showed an abscess in the left leg (15 cm×5 cm×6 cm). The culture of the pyogenic fluids grew *Salmonella Dublin*. He was treated with Meropenem and Vancomycin and underwent incision and drainage of the abscess in the left leg. He discharged after comprehensive therapy.

### Third admission

**Fig. 4:**
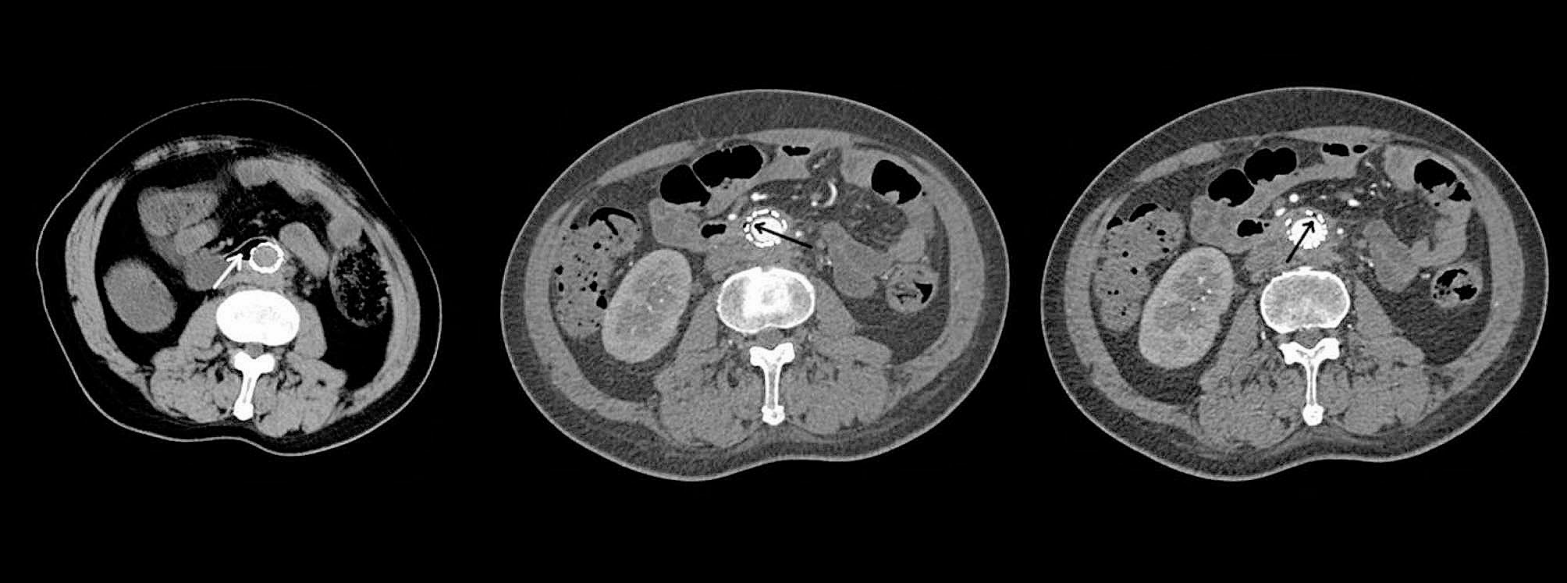
The left image shows the deficiency of soft tissues surrounding the stents. The middle and right image depict intra-stent mural thrombosis and small air bubbles

**Fig. 5:**
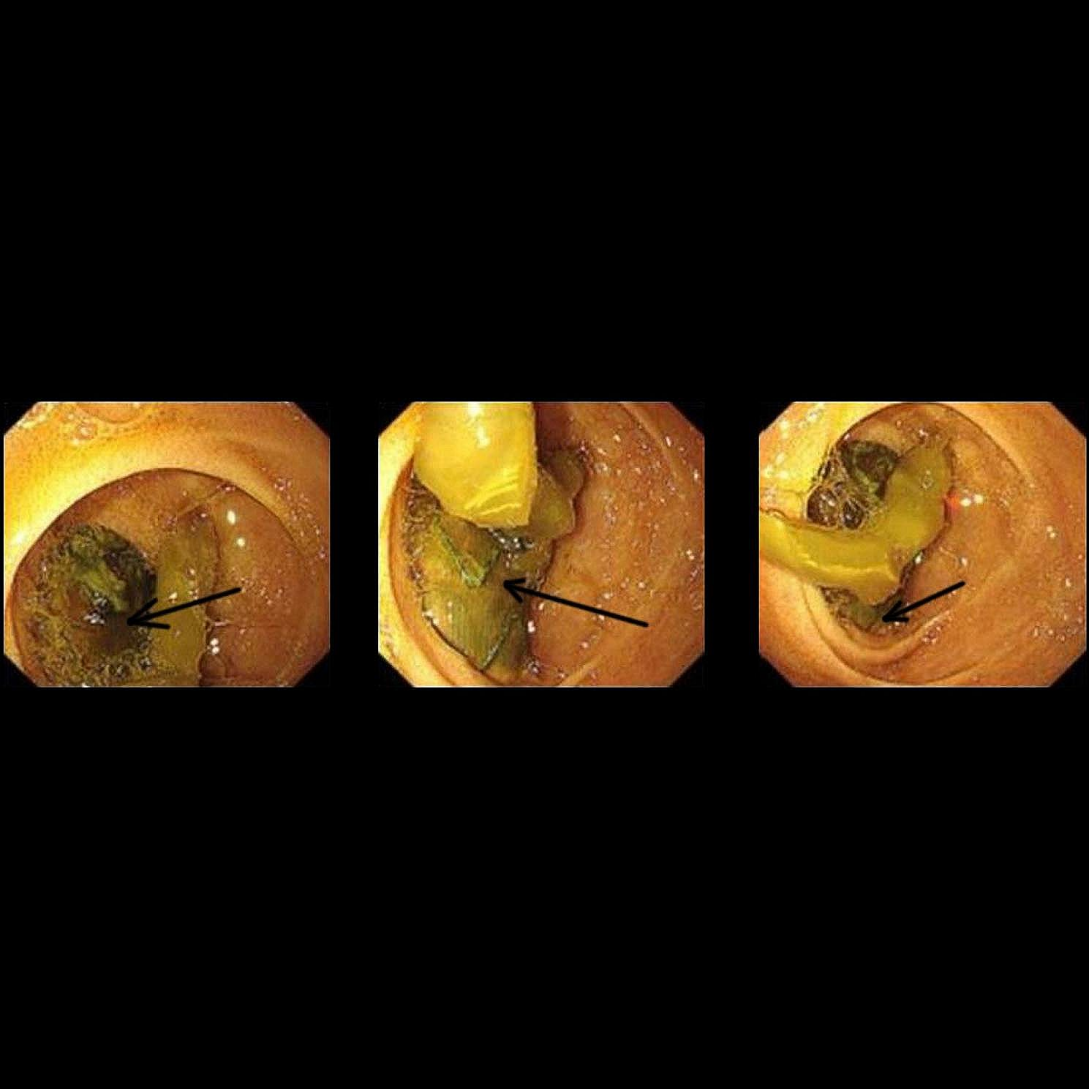
Endoscopy reveals a large duodenal ulcer with visualization of graft material in the third portion of the duodenum

**Fig. 6:**
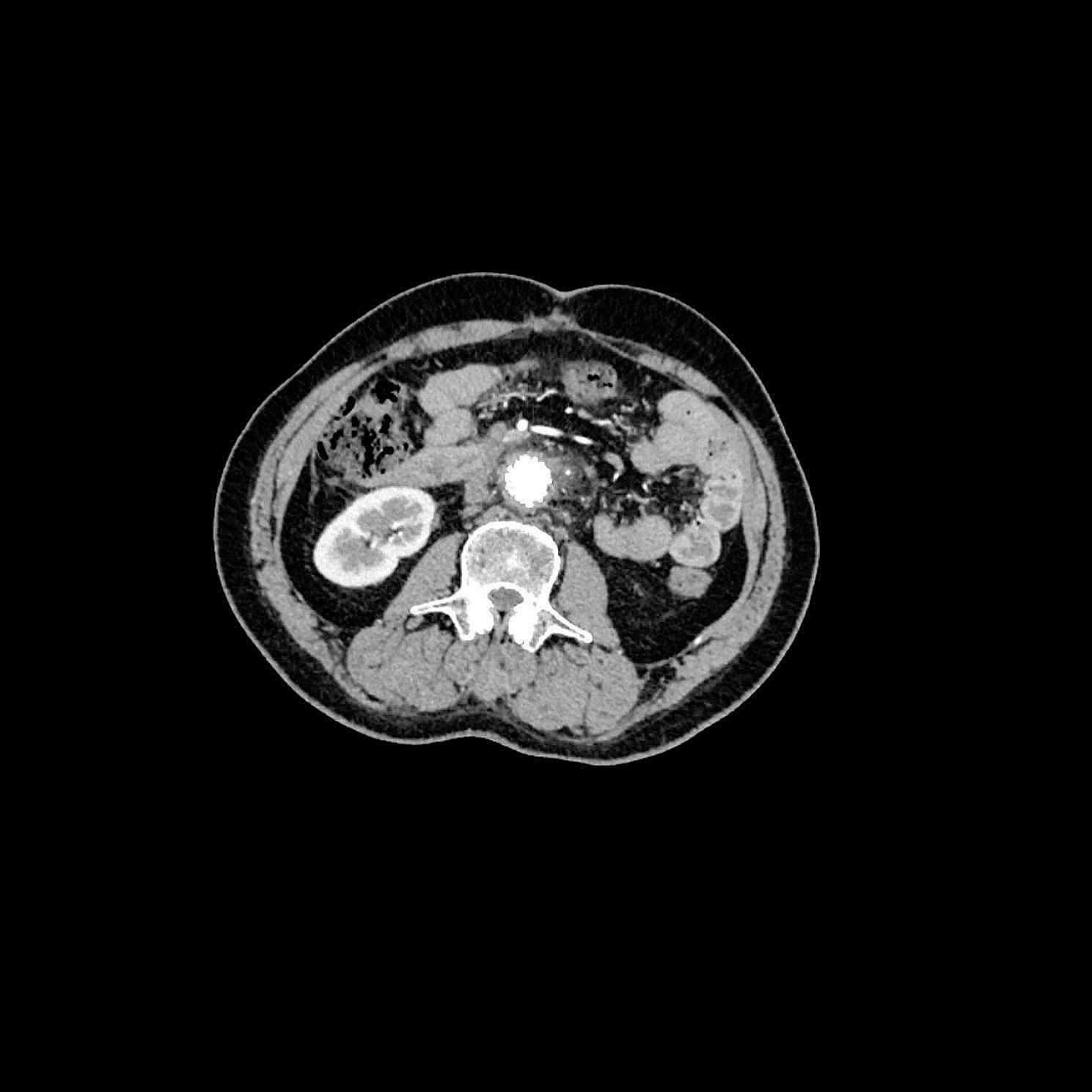
The CTA performed half a year after the operation

The patient was readmitted on August 15th, 2022, with complains of right low extremity pain, recurrent fever for the past three days, and abdominal pain for one day. Physical examination revealed tenderness, rebound tenderness, and muscular tension throughout the abdomen, with increased local skin temperature in the right lower extremity. Blood cultures grew *Enterococcus faecium* and *Escherichia coli*. Sepsis was treated with Meropenem and Vancomycin. CTA showed intra-stent mural thrombosis and small air bubbles, with no sign of leakage. The stent marginbecame adherent to the third portion of the duodenum (Fig. 4). Ultrasonography revealed an abscess in the right planta (4 cm×0.8 cm×1.7 cm). These bacteria were derived from the intestinal tract, suggesting a communication between the intestinal tract and the circulatory system. Esophagogastroduodenoscopy (EGD) revealed a large duodenal ulcer with visualization of graft material in the third portion of the duodenum, indicating an abdominal aorta-duodenal fistula (Fig. 5). According to the MAGIC Classification [2], the diagnosis of aortic graft infection can be made if one major criterion (such as fistula development) and two other criteria from different categories (e.g., suspicious peri-graft findings on radiology, positive blood cultures with no apparent source except aortic graft infection) are met. The patient was transferred to vascular surgery department for in situ abdominal aortic repair, stent fixation, duodenectomy of the third portion, and jejunoduodenostomy. During the operation, the stents and surrounding tissues were soaked in Rifampicin injection for approximately one hour to reduce the risk of post-operation infection. The stents were refastened to the abdominal aorta using vascular sutures and wrapped with an omental pedicle flap. Amoxicillin and Clavulanate Potassium were used for preventing recurrent infection. A year and a half after the operation, the patient was free of infection symptoms, and his infective indices were normal. CTA performed on February 2nd, 2023, showed that the stents was wrapped by soft tissues, with no leakage of contract agent (Fig. 6).

## Discussion and conclusions

In this case, the fistula was secondary to endovascular aneurysm repair performed 2 years ago. CTA and EGD were necessary for defining ADF. Signs such as peri-graft or intra-graft gas, effacement of the intervening fat plane, disruption of the aorta, adjacent inflammatory stranding, and intravasation of contrast into the bowel lumen or peri-graft space favor the diagnosis of ADF [3, 4]. Among patients with HIV-associated infective native aortic aneurysm, the most common microbiological pathogens were Treponema pallidum (31%) and Salmonella spp. (26%) [5]. According to a systematic analysis for the global burden of non-typhoidal Salmonella invasive disease, the mean case fatality among those living with HIV was 41.8% compared with 12.0% among those without HIV [6]. CD4 T-cells, which are decreased in patients with HIV, are crucial for immunity to Salmonella infection [7]. The relationship between HIV infection and vascular structure and function was ambiguous. Previous studies have found that HIV infection can aggravate arterial stiffness, atherosclerosis, and endothelial disfunction though HIV-related inflammation and immune activation, increasing reactive oxygen species production and reducing nitric oxide bioavailability [8–12]. However, there was large heterogeneity between HIV and vascular pathologies in previous reviews, and the results were often contradictory [13, 14]. In our case, the comorbidity, AIDS, aggravated the infection and may accelerate vascular damage, expanding the extent of ADF. Moreover, there was a vicious circle between vessel erosion and gut bacteria translocation. Bacterial translocation has been associated with infection of distant tissue and sepsis morbidity [15]. The ability to disseminate to and infect distal organs was a characteristic property of Salmonella infection [16]. At the second admission, blood cultures grew *Enterococcus faecium*, *Salmonella*, and *Streptococcus anginosus* and the culture of pyogenic fluids grew *Salmonella Dublin*. These reasons might partly explain the recurrent sepsis and lower extremity abscess in this case. Salmonella has been confirmed to cause vascular wall damage, aortic grafts infection, and ADFs [17–21]. Thus, we suspected that the Salmonella sepsis facilitated the formation of SADF in this patient.

For patients with aortic endograft infections, surgical treatment was a better option compared with conservative management [22]. After considering the high risk of hemorrhage and the significant incision required for axillary-bifemoral bypass surgery, we decided, following discussions with the patient and his family members, to opt for in situ abdominal aortic repair. According to previous studies [23–25], soaking prosthetic grafts with rifampicin might inhibit the growth of microorganisms, and reduce the recurrence of infection. Moreover, the course of antibiotic treatment was ambiguous among patients with evident infection, and some researchers advocated that the course between six weeks to a lifetime [18, 26, 27]. Considering the recurrent pre-operative infection and the comorbidity (AIDS), this patient was concepted for long-term antibiotic treatment.

In summary, the initial clue of SADFs could be recurrent infections of non-typhoidal Salmonella and gut bacteria, even in the absence of apparent gastrointestinal bleeding. Patients with immune deficiencies are susceptible to Treponema pallidum and Salmonella infections, which could exacerbate the development of SADFs.

## Data Availability

All data generated or analyzed during this study are included in this published article.

## Abbreviations

SADFs: Secondary Abdominal Aorta-Duodenal Fistulas
AIDS: Acquired Immune Deficiency Syndrome
CTA: Computed Tomography Angiography
EGD: Esophagogastroduodenoscopy
